# 

**Published:** 1886-12-04

**Authors:** 


The Hospital.

An Institution, Family, and Parochial Journal of Hospitals, Asylums, and all Agencies for the Care of the Sick, Criticism and News.

SATURDAY, DECEMBER 4TH, 1886.

### NOTICE TO CORRESPONDENTS.

All correspondence must be written on one side of the paper only.
We cannot undertake to return MSS. not used.
**Communications**, whether intended for insertion or for private information, must be authenticated by the names and addresses of the writers, not necessarily for publication.
**Local papers** containing reports or news paragraphs **should be marked.**
It is especially requested that early intelligence of **local events of interest**, or which it may be thought desirable to bring under notice, may be sent direct to the Editor.
Communications respecting editorial matters should be addressed to the Editor, Norfolk House, Norfolk Street, Strand, London, W.C.

### BUSINESS COMMUNICATIONS AND ADVERTISEMENTS.

All communications concerning business matters, non-delivery of THE HOSPITAL, etc., should be addressed to the Manager, at the Publishing Offices, 30 and 32, Sardinia Street, Lincoln's Inn Fields, London, W.C.
**Cheap Advertisements** of the "Wanted" class, including those for situations, of Twenty-four Words or under, are charged 1*s*. an insertion, or three insertions for 2*s*. 6*d*., but must be paid for in advance. Each additional line of Eight Words is 4*d*.
**Employers requiring assistants** are charged for one insertion of Twenty-four Words or under, 2*s*. 6*d*., or 6*s*. for three insertions. Each additional line of Eight Words is 1*s*.
**Advertisements** for insertion in the weekly issue of THE HOSPITAL **must be delivered** at the Publishing Offices **not later than 11 a.m. on Wednesdays.**

### The In- and Out-Patients' Hospital Diary.

The following forms the first instalment of our combined In- and Out-Patients' Diary, and comprises twenty-three General, the Cancer and the Children's Hospitals in the Metropolis. The Diary will be continued week by week, so as to be completed in each monthly part.

### I.—GENERAL HOSPITALS.

| Hospitals and Terms of Admission. | Physicians, Surgeons, and Medical Officers, with Days and Hours of Attendance. |
|---|---|
| Charing Cross Hospital, West Strand, W.C. Sec., Mr. A. E. Reade<br>Urgent cases are immediately admitted as in-patients. Subscriber's letter expected in other cases<br>Out-patients admitted without subscriber's letter first time; for continued attendance they must be obtained from the Secretary | *In-Physicians*, Drs. Pollock, M. Th.; Green, W. S.; Bruce, Tu. F. Hour, 1.30<br>*In-Surgeons*, Messrs. Barwell, M. Th.; Bellamy, Tu. F.; Bloxam, W. S. Hour, 2<br>*Out-Physicians*, Drs. Wilcocks, M.Th.; Abercrombie, Tu. F.; Lubbock, W. S.; Murray, W. S. Hour 1.30<br>*Out-Surgeons*, Messrs. Cantlie, M.Th.: J. H. Morgan, Tu. F.; Stanley Boyd, W. S. Hour, 1.30 |
| "Dreadnought" Hospital (Seamen's Hospital Society), Greenwich, S.E. Sec., Mr. W. T. Evans<br>Entirely free to seamen and to all accident cases | *In- and Out-Physicians*, Drs. Curnow, Tu. Th. S.; H. T. Griffiths, M.W.F. Daily, between 9 and 3<br>*In- and Out-Surgeons*, Messrs. W. J. Smith, G. R. Turner. Daily, between 9 and 3 |
| French Hospital, 10, Leicester Place, Leicester Sq., W.C. Sec., Mr. E. Rimmel<br>Free to urgent cases and to all foreigners speaking French | *In-Physician*, Dr. Vintras, Tu. S. 10<br>*In-Surgeons*, Sir W. MacCormac, Tu. 9; Mr. J. Késer, W. F. 10<br>*Out-Physician*, Dr. Vintras, Tu. S. 10<br>*Out-Surgeons*, Messrs. H. de Méric, M. Th. 10; J. Késer, W. F. 10 |
| German Hospital, 113, Dalston Lane, E. Sec., Mr. C. Feldmann<br>Free to natives of Germany, and others speaking German, and to all accidents and emergencies. Others by Governor's letters | *In-Physicians*, Drs. Weber, M. T. 2; Port, Tu. F. 9 a.m.<br>*In-Surgeons*, Drs. Lichtenberg and Burger, W. S. 2<br>*Out-Physicians*, Drs. Tuchmann, Tu. W. 2; Ludwig, M. Th. 2; Keller, Tu. W. F. 2; Philippi, M. Th. F. 2 (*Men*, Tu. Th.; *Women*, M. W. F.) |
| ——— Eastern Branch, 49, Mansell Street, Aldgate, E. | *Out-Physician*, Dr. C. Harrer, Tu. F. 8 to 10 a.m. |
| ——— Western Branch, 259, Oxford Street, W. | *Out-Physician*, Dr. M. Castaneda, M. T. 8 to 10 a.m. |
| Great Northern Central Hospital, Caledonian Road, N. Sec., Mr. W. T. Grant<br>Free to all, without letters of recommendation | *In-Physicians*, Drs. Cholmeley, Cook, and Burnett, M. Tu. W. Th. F. 2<br>*In-Surgeons*, Messrs. W. Adams, W. Spencer Watson, and J. Macready, M. Tu. W. Th. F. 2<br>*Out-Physicians*, Drs. Beale, M. Th. 2; Beevor, Tu. 2, S. 10<br>*Out-Surgeons*, Messrs. Lockwood, M. Th. 2; Makins, Tu. F. 2 |
| Guy's Hospital, St. Thomas's Street, S.E. Supt., Dr. C. J. Steele<br>Patients are admitted, if beds are available, without letters of recommendation, on application at the Hospital. Out-patients free without letters of recommendation | *In-Physicians*, Drs. Pavy, Tu. F.; Pye-Smith, M. Th. S.; Taylor, M. Tu. Th. F. Hour, 1.30<br>*In-Surgeons*, Messrs. Bryant, M. Th. Durham, M. Th. F.; Howse, W. S.; D. Colley, M. Th. Hour, 1.30<br>*Out-Physicians*, Drs. Carrington, W.; Hale White, M.; Goodhart, M. Tu. Th. F. Hour, 12.30<br>*Out-Surgeons*, Messrs. Lucas, Th.; Golding Bird, S.; Jacobson, W.; Symonds, M. Hour, 12.30 |
| King's College Hospital, Portugal Street, W.C. Sec., Mr. T. Mosse Macdonald<br>Accidents and urgent cases free at all times. Other cases by Governors' letters, but where not obtainable, application may be made to the Secretary | *In-Physicians*, Drs. Beale, Duffin, Yeo, Tu. W. F. 1.30; Tu. W. F. S. 2<br>*In-Surgeons*, Mr. Wood, Sir J. Lister, Messrs. H. Smith, Cheyne, W. Rose, Barrow. Daily, 1.30<br>*Out-Physicians*, Drs. Ferrier, J. Curnow, N. I. C. Tirard; *Men*, Tu.Th.; S. 1; *Women*, M. W. F. 1<br>*Out-Surgeons*, Messrs. W. Rose, W. Cheyne; *Men*, Tu. Th. S. 1; *Women*, M. W. F. 1 |
| London Hospital, Whitechapel Road, E. Sec., Mr. A. Haggard<br>Accidents and urgent cases free; out-patients for dental and maternity departments need no recommendation; other cases by subscriber's order. Out-patients are required to attend only once, unless otherwise directed by the Physician or Surgeon | *In-Physicians*, Drs. Mackenzie, Tu. F.; Down, Tu. F.; H. Jackson, M. Th.; Sutton, M. Th.; Fenwick, Tu. F. 1.30<br>*In-Surgeons*, Messrs. Rivington, Tu. F.; Couper, Waren Tay, M. Th.; McCarthy, W.S.; Treves, Tu.F. Hour, 1.30<br>*Out-Physicians*, Drs. Sansom, Th.; M. Ralfe, M. Th.; Turner, Tu. F.; Warner, Tu. F.; Gilbart Smith, W. S. J. Anderson, W. S. Hour, 1.30<br>*Out-Surgeons*, Messrs. Mansell-Moullin, M. Th.; Eve, M. W.; Reeves, Tu. S.; H. Fenwick, Tu.F. Hour, 1.30 |

| Hospitals and Terms of Admission. | Physicians, Surgeons, and Medical Officers, with Days and Hours of Attendance. |
|---|---|
| London Temperance Hospital, Hampstead Road, N.W.<br>For treatment without alcohol. By letter or small payment | *In- and Out-Physicians*, Drs. J. Edmunds, M.; J. J. Ridge, Tu. F. Hour, 1.30<br>*In- and Out-Surgeons*, Mr. A. P. Gould, Th. 2.30 |
| Metropolitan Free Hospital, Kingsland Road, N. Sec., Mr. G. Croxton<br>Admission free to the sick poor, without any letter of recommendation. Urgent cases at all times | *In- and Out-Physicians*, Drs. Drysdale, Tu. F. 12; J. G. Dudley, W. S. 12; Fotherby, M. Th. 12; H. H. Tooth, Tu. F. 9; A. Haig, W. S. 9; A. Davies, M. Th. 9<br>*In- and Out-Surgeons*, Messrs. E. J. Chance, W. S. 9; Goodsall, Tu. F. 9; Walsham, M. Th. 9; A. A. Bowlby, F. 9; S. Paget, Th. 9; C. J. Thompson, daily, 9 |
| Middlesex Hospital, Berners Street, W. Sec. and Supt., Mr. A. O. D. Bartholeyns<br>Accidents and urgent cases are admitted free; all ordinary cases by Governor's or subscriber's letter. Patients are seen for Electro-Diagnosis and treatment on M. and T. at 3, and on F. at 1.30 | *In-Physicians*, Drs. Cayley, M. Th.; Coupland, Tu. F.; D. Powell, M.Th.; Finlay, Tu. F. Hour, 1.30<br>*In-Surgeons*, Messrs. Hulke, M. Th.; Lawson, M. Th.; Morris, Tu. F. Hour, 1.30<br>*Out-Physicians*, Drs. Fowler, Tu. F. 3.30; Biss, M. Th. 1.30; Pringle, W. S. 1.30<br>*Out-Surgeons*, Messrs. Andrew Clark, M. 1.15 (*Men*); Th. 1.15 (*Women*); Pearce Gould, F. 1.30 (*Men*); Tu. 1.30 (*Women*); J. B. Sutton, W. S. 9 |
| Miller Hospital and Kent Dispensary, Greenwich, S.E. | *Physicians*, Drs. T. Creed, C. H. Hartt, G. Willes<br>*Surgeons*, Messrs. T. Moore, J. Poland, E. Clarke |
| North-West London Hospital, 18, Kentish Town Road, N.W. Sec., Mr. A. Craske<br>By subscribers' tickets or payment, but accidents and urgent cases free at all times | *In- and Out-Physicians*, Drs. W. C. Hood, J. Shaw, Edwarde H. Campbell. Daily, 1.30<br>*In- and Out-Surgeons*, Messrs. F. Durham, Collier, Jennings, J. Black. Daily, 1.30 |
| Ospedale Italiano, 41, Queen Square, Bloomsbury. Sec., Sig. L. Bonacina<br>Free to all, but Italians have preference | *In- and Out-Physicians*, Drs. M. Castaneda, Tu. F. 10 to 11; B. Sassella, M.Th. 10 to 11; Mr. J.W. B. Steggall, W. S. 4 to 5 |
| Poplar Hospital, 303, East India Dock Road, E. Sec., Lt.-Col. Feneran<br>Free to accidents and emergencies | *In-Surgeons*, Messrs. F. M. Corner, M. Brownfield, and Resident Surgeons<br>*Out-Surgeons*, Dr. J. D. Grant, W. S.; Mr. T. E. Bowkett, Tu. F. 12 to 2 |
| Royal Free Hospital, Gray's Inn Road, W.C. Sec., Mr. J. S. Blyth<br>Admission free to the sick poor, without any letter of recommendation | *In- and Out-Physicians*, Drs. Cockle, M. Th.; Sainsbury, Tu. F.; West, W. S. Hour, 1<br>*In- and Out-Surgeons*, Messrs. Rose, M. Th.; W. Gant, Tu. F.; Barrow, W. S. Hour, 1 |
| St. Bartholomew's Hospital, Smithfield. Clerk, Mr. W. Cross<br>Admission free, without letter or ticket. Accidents and other urgent cases admitted at any time; ordinary cases of disease daily, between 9 and 10. Patients are seen for Electrical treatment on M. Tu. Th. and F. 1.30 | *In-Physicians*, Drs. Andrew, Church, Gee, Sir D. Duckworth. Daily, 1.30<br>*In-Surgeons*, Messrs. Savory, T. Smith, Willett, Langton, M. Baker. Daily, 1.30<br>*Out-Physicians*, Drs. Hensley, M. Th. 11; Brunton, Tu. F. 11; Legg, W. S. 11; N. Moore, Tu. W. Th. S. 9<br>*Out-Surgeons*, Messrs. Marsh, Tu. F.; 12; Butlin, M. Th. 12; Walsham, W. S. 12; Cripps and Bruce Clarke, daily, 9<br>*Casualty Physicians*, Drs. Davies, Tu. W. F. S.; O. Browne, M. Tu. Th. F.; Nias, M. W. Th. S. Hour, 9 |
| St. George's Hospital, Hyde Park Corner, S.W. Sec., Mr. C. L. Todd<br>Free to accidents and emergencies. Other in-cases by Governor's or subscriber's letter, but, in necessitous cases, this recommendation is not strictly insisted on. Letters are not required for out-patients. Vaccination on Th. at 11 | *In-Physicians*, Drs. Wadham, M. F.; Dickinson, M. Tu. Th. F.; Whipham, Tu.W. S.; Cavafy, Tu. S. Hour, 1<br>*In-Surgeons*, Messrs. Holmes, M. Th, F. 1, W. 1.30; Rouse, M. Th. F. 1, W. 1.30; Haward, Tu. Th. S. 1, W. 1.30<br>*Out-Physicians*, Drs. Ewart, M. F.; Owen, Tu. S. Hours, 10.30 to 12<br>*Out-Surgeons*, Messrs. Bennett, M. F.; Dent, Tu. S. Hours, 10.30 to 12<br>*Men*, F. S.; *Women*, M. Tu. |

### THE CHILDREN'S CORNER.

UBIQUE suggests that some mathematical puzzles would be amusing. I have already anticipated her wish, and have prepared a selection which I propose to set from time to time. "Mother," who dates from The College, Glasgow, sends me two or three clever conundrums, which, she thinks, may amuse some of the little sick ones who read THE HOSPITAL. "Some of us," she adds, "try to guess the puzzles and conundrums in our weekly HOSPITAL, but alas, find ourselves too stupid to guess any but the very easiest; the amusement, however, is just the same, and we are so surprised at the cleverness of the successful ones. . . . We like THE HOSPITAL very much here, and think it will do good."

### OUR PASTIMES.

**Children's Quarterly Prize Competition.**

| | |
|---|---|
| Began .. .. .. .. | 2nd Oct., 1886 |
| Ends .. .. .. .. | 25th Dec., 1886 |

PRIZES.

| | |
|---|---|
| 1st .. .. .. .. | Thirty Shillings |
| 2nd .. .. .. .. | Twenty " |
| 3rd .. .. .. .. | Ten " |
| 4th .. .. .. .. | Five " |

will be awarded to the four competitors who shall have sent in, during the thirteen weeks, the largest number of correct answers to our pastimes.

**Tenth Competition.**

No. 49. What two animals went into the ark provided with their toilet necessaries?

No. 50. Two "i's," two "e's," an "m," and a "d,"
Add thereto an "n" and a "c,"
Put them together quite orderly
And spell, please you, the word to me.

No. 51. Two straight lines, a circle, two semi-circles on a straight line, a triangle on two legs, two semi-circles and a circle give a word of seven letters. What is it?

No. 52. My *first's* a piece of jutting land,
My *second's* heard in caves on strand,
My *third* a stupor doth impose,
My *fourth* good order ever shows,
My *fifth* a famous shield describes,
My last's a useful beast to northern tribes.
Initials give a discoverer great,
While the finals do his profession state.

No. 53. A man undertook to turn into a rhyming couplet a sentence which did not contain two rhymes, and without changing a single word. The sentence given was "There was an old woman, and she was as deaf as a post." How did he do it?

No. 54. Said a gentleman to a young lady:—
My *first* is myself in a very short word,
My *second's* a puppet, and you are my *third*.
The young lady guessed the compliment; can you, my little friends, guess it also?

**Results of Eighth Competition.**

No. 38. This was guessed by every competitor, but for the sake of other readers, I give the solution here:

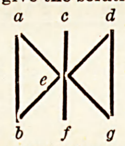

The direction to take is *c* to *e*, *e* to *a*, *a* to *b*, *b* to *e*, *e* to *d*, *d* to *g*, *g* to *e*, and *e* to *f*.

No. 39. Only Tina is successful here. Retsibsi thinks that the swift entered quickest and the sloth slowest, but he is not right. The elephant went slowest into the ark because he had his *trunk* to carry, while the spider went quickest, for he doubtless *took a fly!*

No. 40. Palindromes are exceedingly difficult to make, and hence I am not surprised that Ubique and Retsibsi have alone made sentences which will read backwards and forwards the same. Ubique sends a reply which Napoleon is said to have made when at St. Helena, "Able was I ere I saw Elba," while Retsibsi writes "Lewd I did live and evil did I dwel' but this is unfortunately imperfect in the last word. I have received several palindromic words such as rotator, sexes, Hannah, deed, Eve, eye, noon, did, dad, etc.

No. 41. Alsie, who is always *au fait* at Latin puzzles, and T.K. divide honours here. "Mea, mater, sus malum est," reads "Run, mother, the sow is eating the apple."

No. 42. The gardener might have planted the trees in either of the two following orders:

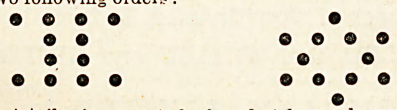

No. 43. A tailor is never to be found at home, because he is always *cutting out!*

All right—None.
Five right—Tina.
Four right—T. K., Jimmy, Retsibsi, Alsie.
Three right—Little Mary, Ellie, Ubique, Bismarck.
Two right—Diosota, Hetty, True Blue.

Answers, having the actual name and address (to which a pseudonym may be added for publication), must have "The Children's" Coupon attached (see advertisement pages), addressed to the "PUZZLE EDITOR," THE HOSPITAL, Norfolk House Norfolk Street, Strand, W.C., not later than Thursday, 9th December. Results will appear in our issue of the 18th December.

### "THE HOSPITAL" CHARITY BOX.

OUR "Charity Box" is intended to afford a little practical help to the hospitals. Each week we propose a word for "anatomical dissection," and the first 150 competitors who extract the largest number of words from it receive the following prizes:—

| | |
|---|---|
| 1st .. .. .. | One Guinea. |
| 2nd .. .. .. | Half a Guinea. |
| 3rd .. .. .. | Five Shillings. |

The above Prizes will be repeated for every additional 150 competitors who may enter each week

TENTH COMPETITION.

Compose the largest number of words you can from "MEDICINE."

*Observe:* Proper names, abbreviations, foreign, Latin, and obsolete words are barred.

Repetitions of similarly spelt words and of letters (unless they repeat in the word) are disallowed.

Plurals allowed, but not the possessive in 's or s'.

Prefixes and affixes do not count independently of words.

Write only on one side of the paper, and total your words.

Append your real name and address, adding whether Mr., Mrs., or Miss, etc.

All replies, addressed "Charity Box," THE HOSPITAL, Norfolk House, Norfolk Street, W.C., must arrive by Friday, Dec. 10th. Results will appear Saturday, Dec. 18th.

*Every Competitor is required to cut out and enclose with his list the "Charity Box" Coupon* (see advertisement pages), *and a shilling entrance fee* (postal orders only, *crossed Lloyd's, etc., Banking Company, Limited*, THE HOSPITAL *Account.*)

The proceeds resulting from each "Charity Box" competition will be funded until the end of December 1886. The total sum available for distribution, and the names of the hospitals selected for donations, will appear on Saturday, January 8, 1887 The distribution of the proceeds will be at the Editor's sole discretion.

RESULTS OF EIGHTH COMPETITION

The "Surgeon" Competition has resulted as follows:—

| | Accepted Score. |
|---|---|
| Mr. E. E. Bruce, 15, Holbeck Road, Brixton, S.W. .. .. .. .. .. .. .. .. | 121—1st Prize. |
| Mr. W. H. Sanderson, Berry Wood, Northampton .. .. .. .. .. .. .. | 119—2nd Prize. |
| Miss Corbishley, The Byrons, Sutton, Macclesfield .. .. .. .. .. .. | 118—3rd Prize. |

The prizes have been forwarded to the winners.

**Special Notice.**

In the interest of non-successful competitors, we have decided not to award the First Prize more than once to a winner in any one quarter. In the event of a competitor again making the highest accepted score, he will be placed in the second place, and if a third time highest, in the third place.

As so many of our correspondents continue to ask us to publish at least one successful list of words, we have decided to comply with their request, but, in consequence of the pressure on our space, the list must be a small one. We have therefore decided to print the highest accepted list from "Doctor," set last week. Some of our correspondents seem to doubt the possibility of anyone making so many genuine English words as the published results show. We hope therefore that by the publication of the promised list we shall dissipate the doubt which, despite our assurances, some of our unsuccessful competitors appear still to entertain.

**Notices to Competitors.**

Miss A. Clift.—We disallow the possessives.

Mr. T. H. Forster.—Some of your words, such as "Oh," "negroes," etc., cannot be obtained from "Surgeon." See above answer.

Miss Fryer.—Prefixes are barred.

Mr. Charles W. Sayer.—The Entrance Fee must be repeated each time you compete.

Mr. J. Halsey.—It would, we think, be unwise to ask our readers to take any one dictionary. The same dictionary would probably not be at the command of everyone. If it were, and every competitor searched it with equal care, there would be an all round "tie."

Mrs. Bedingfeld.—You will doubtless be pleased to see that we have agreed to publish the highest accepted score from "Doctor." We hope you will now ask your friends to compete.

Mizpah.—Please see "Special Notice." Proper nouns are names appropriated to individual things, places, and persons, and such words we bar.

Toby.—We have now stated in our Rules that plurals are allowed. The choice of dictionaries we must leave entirely in the hands of the competitors.

